# Novel Inhibitory Effects of Glycyrrhizic Acid on the Accumulation of Advanced Glycation End Product and Its Receptor Expression

**DOI:** 10.1007/s13659-014-0044-0

**Published:** 2014-11-05

**Authors:** Hong Sheng Cheng, Joana Magdelene Xiao Fang Kong, Athena Xin Hui Ng, Weng Keong Chan, So Ha Ton, Khalid Abdul Kadir

**Affiliations:** 1 School of Science, Monash University Malaysia, 46150 Bandar Sunway, Selangor, Malaysia; 2 School of Medicine and Health Sciences, Monash University Malaysia, 46150 Bandar Sunway, Selangor, Malaysia

**Keywords:** Metabolic syndrome, Receptor for advanced glycation end product, Licorice, High-fat/high-sucrose diet

## Abstract

Beneficial effects of glycyrrhizic acid (GA), a bioactive extract of licorice root, in the prevention of metabolic syndrome have been consistently reported while advanced glycation end products (AGE) and receptor for advanced glycation end product (RAGE) are the leading factors in the development of diabetes mellitus. The aim of this study was to investigate the effects of GA on the AGE-RAGE axis using high-fat/high-sucrose (HF/HS) diet-induced metabolic syndrome rat models. Twenty four male Sprague–Dawley rats were randomly assigned into three groups for 4 weeks: (1) Group A, normal diet with standard rat chow; (2) Group B, HF/HS diet; (3) Group C, HF/HS diet and oral administration of 100 mg/kg GA per day. The results showed that HF/HS diet elevated the fasting blood glucose level and insulin resistance index which was prevented by GA supplementation. GA treatment significantly lowered the circulating AGE independent of its glucose-lowering effect. HF/HS diet also triggered RAGE upregulation in the abdominal muscles while GA administration downregulated RAGE expression in the abdominal muscles, aorta and subcutaneous adipose tissues. In conclusion, HF/HS diet could cause glucose intolerance, insulin resistance and upregulation of RAGE expression while GA ameliorated the metabolic dysregulation besides exhibiting inhibitory effects on the AGE-RAGE axis.

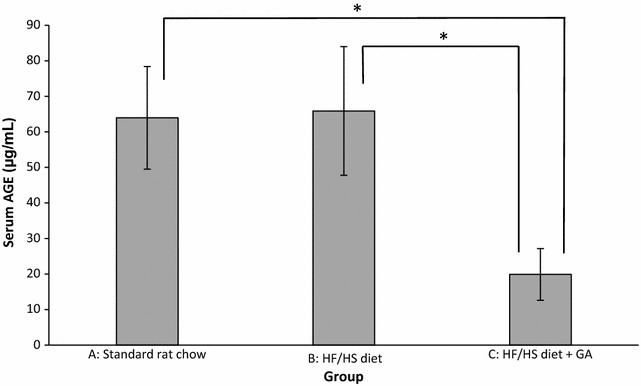

## Introduction

Advanced glycation end product (AGE) is an irreversible product resulted from non-enzymatic glycation of reducing sugars like glucose and fructose to amino acid groups in proteins and lipids, followed by the formation of Schiff’s bases, Amadori products and eventually AGEs via a series of rearrangement and oxidation reactions [1]. The glycation pathway is known as Maillard reaction. Some of the most abundant AGEs found in tissue proteins include *N*-ε-(carboxymethyl)lysine, *N*-ε-(carboxylethyl)lysine, pentosidine and pyrraline. Aside from Maillard reaction, AGE formation can also be accelerated by other pathways, notably glucose autooxidation, lipid peroxidation and polyol pathway [2, 3].

Advanced glycation end product and the receptor for advanced glycation end product (RAGE) are also increasingly recognized as one of the leading factors to the onset of diabetic vascular complications. In patients with type 2 diabetes mellitus, the level of circulating AGE was found to have significantly escalated, implying that the rate of glycation reaction is highly concentration-dependent [4]. The accumulated AGEs can interact with RAGE which initiates multiple signaling cascades and subsequently activates a regulatory protein for inflammatory and immune responses, nuclear factor-κB (NF-κB) [5]. This links the AGE-RAGE axis to various pathological manifestations in the blood vessels such as proinflammation, prothrombosis and increased leucocyte adhesion that contribute to the development of endothelial dysfunction [1]. Moreover, RAGE expression is also upregulated by NF-κB [6]. As a result, the entire RAGE-NF-κB inter-activation becomes a self-perpetuating vicious cycle fuelled by AGE ligand binding and transforms momentary inflammatory response to an exaggerated form of sustained, chronic inflammation at the endothelium [7].

Despite the AGE- and RAGE-facilitated diabetic vascular injury, their roles and changes during the pre-diabetic state or in metabolic syndrome are under-investigated. In this context, metabolic syndrome has been the main research focus of our laboratory. We have managed to establish diet-induced metabolic syndrome rodent models using high-fat diet [8], high-sucrose diet [9] and high-fat/high-sucrose (HF/HS) diet [10]. The combined diet was used in the present study as it is similar to the unhealthy dietary pattern of people nowadays and hence, providing higher magnitude of generalizability beyond the scope of this study. While trying to understand the metabolic syndrome, we also actively seek for potential therapeutic products from natural resources. One such bioactive compound is glycyrrhizic acid (GA), which is a major constituent found in the root of the genus *Glycyrrhiza* (Leguminosae) or licorice plants. To elucidate, GA is a non-selective inhibitor of 11β-hydroxysteroid dehydrogenase (11β-HSD) whose function is to catalyse the inter-conversion of active and inactive glucocorticoids [11]. Inhibitory effect of GA on 11β-HSD type 1 isoform helps to normalise the insulin sensitivity and the activity of gluconeogenic enzymes which are otherwise, deranged by the unhealthy diet [9]. In addition, GA is also a potent agonist for peroxisome proliferator-activated receptor (PPAR)-γ [12, 13]. PPAR-γ is markedly expressed in adipocytes and is responsible for adipocyte differentiation and lipid storage. GA-facilitated PPAR-γ upregulation is linked closely to improvements in glucose tolerance, insulin sensitivity, lipid profile and ectopic lipid deposition [12–14].

Considering the predominant roles of the AGE-RAGE axis in the progression of diabetes mellitus, it seems pertinent to look at AGE accumulation and RAGE expression in the early stage of the metabolic syndrome. Furthermore, it is also of great research interest to examine the potential inhibitory properties of GA on AGE and RAGE after knowing the beneficial effects of GA on ameliorating metabolic abnormalities. Thus, the objective of the present study was to investigate the effects of orally-administered GA on the AGE-RAGE axis of rats fed on HF/HS diet. Aside from circulating AGE and RAGE expression in multiple tissues, glucose tolerance and insulin sensitivity were also assessed to give a better idea of the metabolic state of the rats.

## Results and Discussion

### GA Prevented Diet-induced Glucose Intolerance and Insulin Resistance

**Fig. 1:**
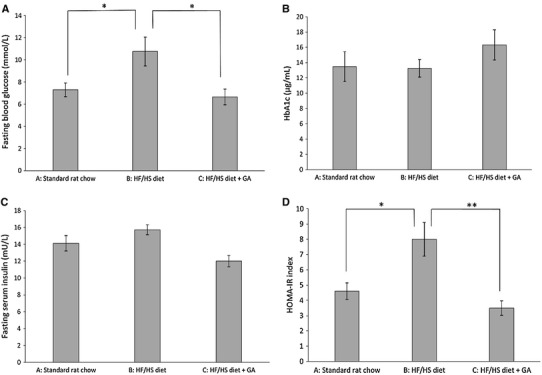
Mean (±SEM) **a** fasting blood glucose concentrations, **b** HbA1c, **c** fasting serum insulin, **d** HOMA-IR of the rats in Groups A, B and C at the end of the four-week treatment (*p < 0.05 between groups; **p < 0.01 between groups). *GA* glycyrrhizic acid, *HbA1c* glycated haemoglobin A1c, *HF/HS* high-fat/high-sucrose, *HOMA-IR* homeostatic model assessment-insulin resistance

**Fig. 2:**
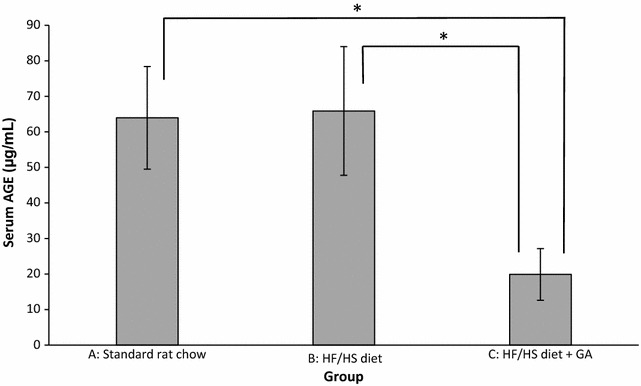
Mean serum AGE concentrations (±SEM) of rats in Groups A, B and C at the end of the four-week treatment (*p < 0.05 between groups). *GA* glycyrrhizic acid, *HF/HS* high-fat/high-sucrose

The mean fasting blood glucose levels (±SEM) of rats from Groups A, B and C were 7.30 ± 0.62, 10.76 ± 1.30 and 6.66 ± 0.71 mmol/L respectively (Fig. 1a). The blood glucose level of Group B rats was significantly higher than that of the other two groups (*p* < 0.05) whereas there was no difference in blood glucose levels between Groups A and C (*p* > 0.05). This shows that consumption of HF/HS diet elevated the circulating glucose, causing the rats to be hyperglycaemic while oral administration of GA helped to prevent the escalation and maintain the blood glucose within the normal range. However, there was no statistical significance in the mean HbA1c concentrations between groups (*p* > 0.05) (13.48 ± 1.95, 13.25 ± 1.15 and 16.32 ± 1.97 µg/mL for Groups A, B and C respectively) (Fig. 1b). Similarly, there was also no significant difference in the AGE level between the groups on normal diet and HF/HS diet (without GA treatment) (Fig. 2) even though the fasting blood glucose of the rats feeding on HF/HS diet was significantly higher. The inconsistencies suggest that the diet-induced hyperglycaemia might not be chronic enough to elevate HbA1c and circulating AGE levels.

The mean fasting serum insulin concentrations (±SEM) of rats from Groups A, B and C were 14.12 ± 0.92, 15.72 ± 0.59 and 12.01 ± 0.68 mU/L respectively (Fig. 1c). There was no significant difference in the insulin concentrations between groups (*p* > 0.05) although the rats on HF/HS diet appeared to have higher level of circulating insulin than rats from the other two groups. HF/HS diet and GA have substantial impacts on the development of insulin resistance. Group B rats had the highest mean homeostatic model assessment-insulin resistance (HOMA-IR) value (±SEM), 8.00 ± 1.11 which was significantly higher than that of Group A (4.61 ± 0.54) (*p* < 0.05) and Group C (3.50 ± 0.47) (*p* < 0.01) (Fig. 1d). The high HOMA-IR value indicates that rats on HF/HS diet for four weeks had develop insulin resistance in comparison to those on a normal diet. There was no difference in HOMA-IR indices of Groups A and C rats (*p* > 0.05).

The normal fasting blood glucose level of rats is comparable to that of humans, which ranges from approximately 5 to 7.5 mmol/L [15]. In this experiment, the fasting blood glucose of the rats on only HF/HS diet was 10.76 ± 1.30 mmol/L, indicating that the rats were hyperglycaemic. Although hyperinsulinaemia was not manifested, the hyperglycaemic status was accompanied by development of insulin resistance as evidenced by significantly higher HOMA-IR when compared to the rats on standard rat chow. This shows that the rats on HF/HS diet are deemed adequate to be used as the metabolic syndrome models for research purpose. The deleterious impacts of unhealthy diet high in sucrose and/or saturated fat on metabolism have been consistently reported not only in animal studies [16–18] but also in clinical studies [19, 20]. The result of this study is in line with these studies.

By using GA as a supplement, the HF/HS diet-associated metabolic abnormalities were effectively prevented. This observation is in concordance with the reported findings from our laboratory which showed that GA treatment could ameliorate metabolic symptoms like hyperglycaemia, insulin resistance and dyslipidemia in the rats given high load of fat [8] and sucrose [9]. GA and its derivatives are known to be PPAR-γ agonists [21]. Chia et al. [22] also revealed that oral administration of GA was associated with upregulation of PPAR-γ expression in the VAT [22]. In the adipose tissues, PPAR-γ activation is crucial for adipogenesis by regulating expression of genes which are involved in adipocyte proliferation and differentiation as well as in fatty acid metabolism [23]. The PPAR-γ agonistic property of GA may play a key role in restoration of the equilibrium between adipogenesis and lipid influx and so, exerting modulatory effect onto the development of adiposopathy or so-called ‘sick fat’.

In addition, GA is also known to be a non-selective 11β-HSD inhibitor. In the liver, kidney, adipose tissues and skeletal muscles, it has been demonstrated to inhibit the activity of 11β-HSD type 1 whose function is to catalyse the conversion of inactive glucocorticoid to the active form [13]. By doing so, GA can inhibit the glucocorticoid-induced signalling cascades, resulting in the reduction of activity of the rate-limiting gluconeogenic enzyme, phosphoenolpyruate carboxykinase, in the liver and kidney [9]. This also shows that GA can exert effective modulation on gluconeogenesis and restrict hepatic release of glucose into the bloodstream.

### Novel AGE-lowering Effect of GA

GA treatment was able to reduce the level of AGE in the blood stream. The serum AGE level (±SEM) of the rats on GA was 19.90 ± 7.27 µg/mL, which was the lowest in comparison to Groups A (63.95 ± 14.44 µg/mL) and B (65.88 ± 18.10 µg/mL) rats (Fig. 2). The differences were statistically significant between Groups A and C and between Groups B and C (*p* < 0.05), There was no significant difference between the rats on normal diet and those on HF/HS diet (*p* > 0.05). Based on the results, it is evident that GA could reduce the level of circulating AGE significantly. This AGE-lowering effect of GA seems to be independent from its anti-hyperglycaemic effect because the GA-treated rats exhibited comparable fasting blood glucose and HbA1c levels to those on normal diet. Previous studies have reported that GA and its metabolites can reduce early glycation products, intermediates in glycation pathway and AGE [24, 25], but the inhibitory effects cannot be differentiated from its glucose-lowering effect. Therefore, the observation in the present study provides the very first piece of evidence for AGE-lowering effect of GA which is independent from its anti-hyperglycaemic effect.

In this context, it is postulated that GA may enhance the efficiency of AGE detoxification and disposal. The underlying mechanism may be related to its ability to stimulate PPAR-γ expression and activation because some of the PPAR-γ agonists like pioglitazone and rosiglitazone under the pharmacological class of thiazolidinedione have been demonstrated to inhibit AGE accumulation in clinical studies [26, 27]. Along with the decrease in AGE level, the level of soluble RAGE (sRAGE) significantly increased [26, 27]. A sRAGE is a circulating receptor for AGE which is similar to the full-length membrane-bound RAGE without the transmembrane and cytoplasmic signalling domains [28]. The distinctive structure of sRAGE makes it an excellent candidate as a decoy and a competitive inhibitor for its membrane-bound isoform to scavenge circulating AGEs and aid in AGE detoxification [29].The PPAR-γ agonistic properties of thiazolidinedione seems to be closely associated to sRAGE level, suggesting a putative regulatory role of PPAR-γ on sRAGE expression. Hence, since GA also possesses PPAR-γ-activating characteristic, it may inhibit AGE accumulation by enhancing scavenging and clearance of AGEs through sRAGE upregulation.

### GA Normalised Diet-induced RAGE Overexpression

**Fig. 3:**
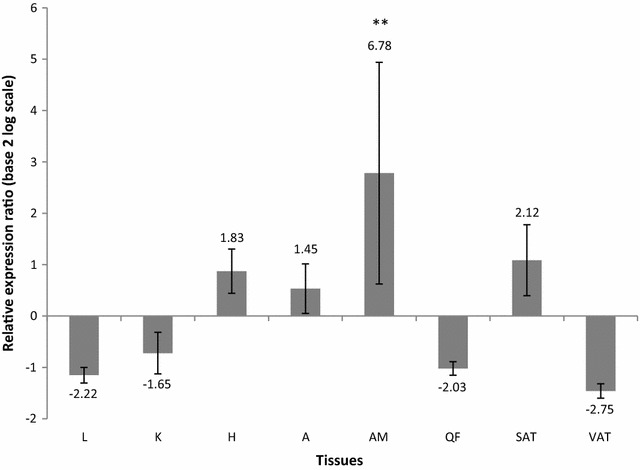
Relative expression ratios (±SE) of RAGE in different tissues in base 2 log scale using BAC as the endogenous reference, Group A (rats on standard rat chow) as the calibrator and Group B (rats on HF/HS diet) as the target (**p < 0.01 between groups). The *data labels* indicate the actual relative expression ratios (in fold) with *positive values* denoting upregulation while *negative values* denoting downregulation. *L* liver, *K* kidney, *H* heart, *A* aorta, *AM* abdominal muscle, *QF* quadriceps femoris, *SAT* subcutaneous adipose tissue, *VAT* visceral adipose tissue

The relative RAGE expression ratios of Group B to Group A rats in different tissues are shown in Fig. 3 in base 2 log scale. RAGE expression (±SE) was upregulated in the heart, aorta and subcutaneous adipose tissue (SAT) by 1.83 ± 0.90, 1.45 ± 1.31 and 2.12 ± 1.35 fold respectively, and was downregulated in the liver, kidney, quadriceps femoris (QF) and visceral adipose tissue (VAT) by 2.22 ± 0.29, 1.65 ± 0.92, 2.03 ± 0.26 and 2.75 ± 0.27 fold respectively. However, these differences in RAGE expression did not reach statistical significance (*p* > 0.05). Only in the abdominal muscle (AM), the expression of RAGE of the rats on HF/HS diet was significantly upregulated by 6.78 ± 5.33 fold compared to those on normal diet (*p* < 0.01).

**Fig. 4:**
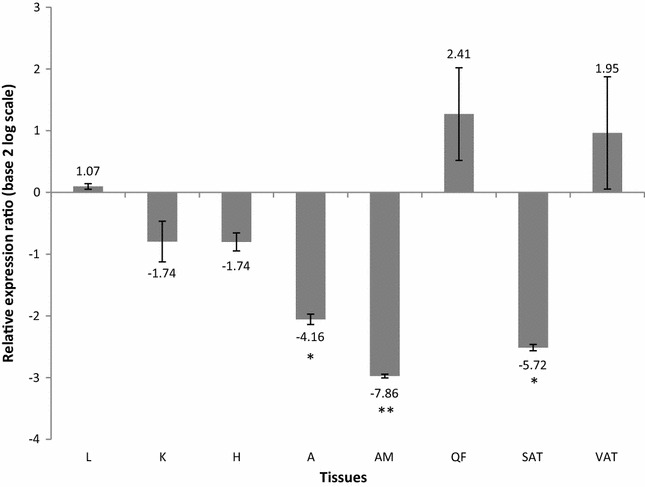
Relative expression ratios (±SE) of RAGE in different tissues in base 2 log scale using BAC as the endogenous reference, Group B (rats on HF/HS diet) as the calibrator and Group C (rats on HF/HS diet and GA) as the target (*p < 0.05, **p < 0.01 between groups). The *data labels* indicate the actual relative expression ratios (in fold) with *positive values* denoting upregulation while *negative values* denoting downregulation. *L* liver, *K* kidney, *H* heart, *A* aorta, *AM* abdominal muscle, *QF* quadriceps femoris, *SAT* subcutaneous adipose tissue, *VAT* visceral adipose tissue

In contrast, when comparing the rats with and without GA treatment, the RAGE expression of Groups C to B rats was significantly downregulated in the aorta, AM and SAT by 4.16 ± 0.17 fold (*p* < 0.05), 7.86 ± 0.085 fold (*p* < 0.01) and 5.72 ± 0.12 fold respectively (*p* < 0.05). The expression was also downregulated in the kidney and heart by 1.74 ± 0.72 and 1.74 ± 0.32 fold respectively but the changes did not reach statistical significance (*p* > 0.05). Conversely, GA-treated rats had their RAGE expression upregulated in the liver, QF and VAT by 1.07 ± 0.51, 2.41 ± 1.43 and 1.95 ± 1.84 fold respectively. The upregulated RAGE expression in the three tissues was not statistically significant (*p* > 0.05). The relative RAGE expression of Groups C to B rats is illustrated in Fig. 4.

**Fig. 5:**
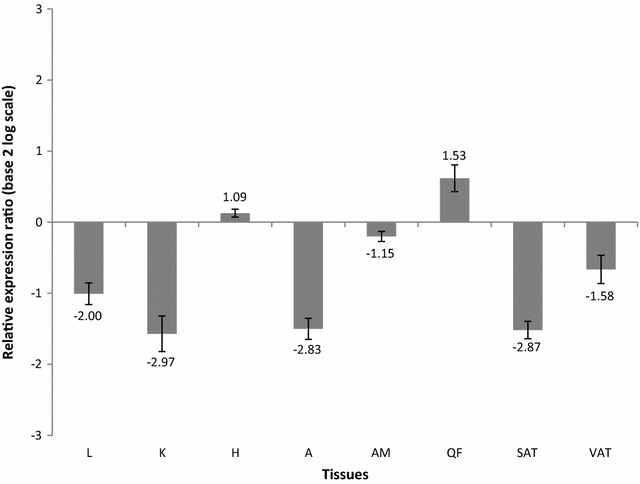
Relative expression ratios (±SE) of RAGE in different tissues in base 2 log scale using BAC as the endogenous reference, Group A (rats on standard rat chow) as the calibrator and Group C (rats on HF/HS diet and GA) as the target. The *data labels* indicate the actual relative expression ratios (in fold) with *positive values* denoting upregulation while *negative values* denoting downregulation. *L* liver, *K* kidney, *H* heart, *A* aorta, *AM* abdominal muscle, *QF* quadriceps femoris, *SAT* subcutaneous adipose tissue, *VAT* visceral adipose tissue

Downregulation of RAGE expression was observed in most tissues of Group C rats in comparison to Group A rat except for the heart and QF where the gene was upregulated by 1.09 ± 0.49 and 1.53 ± 0.47 fold respectively. RAGE expression was downregulated by 2.00 ± 0.30 fold in the liver, 2.97 ± 0.47 fold in the kidney, 2.83 ± 0.28 fold in the aorta, 1.15 ± 0.41 fold in AM, 2.87 ± 0.23 fold in SAT and 1.58 ± 0.47 fold in VAT. Yet, all of the gene regulation did not reach statistical significance (*p* > 0.05), indicating that the RAGE expression of the rats on HF/HS diet with GA treatment did not differ from those on standard rat chow. An illustration of the relative gene expression of Groups C to A rats is shown in Fig. 5.

Generally, RAGE expression was unaffected in some tissues such as the liver, kidney, heart and VAT. This again, supports the postulation that the diet-induced metabolic abnormalities were not chronic enough to trigger significant derangements in the AGE-RAGE axis. What is surprising is that the RAGE expression of the rats on only HF/HS diet was significantly upregulated in AM as compared to the rats on standard rat chow even when the two groups of rats exhibited similar level of circulating AGE level. This is attributable to the multiligand functionality of RAGE. Aside from AGE, RAGE is also known to interact with HMGB1 [30] and S100 protein family [31]. Empirical findings demonstrated that these ligands are also overexpressed and secreted in metabolically-deranged rats [32, 33]. Hence, other ligands might have activated RAGE and triggered resultant signalling cascades and the RAGE overexpression in the AM. Increased RAGE mediated-signalling cascades in the skeletal muscles is linked to pro-inflammation and high oxidative stress [34]. Furthermore, Cassese et al. [35] revealed that RAGE may interact with Src tyrosine kinase, PKC-α and IRS-1 to form a multimolecular complex which desensitises insulin action in the skeletal muscles and causes muscular insulin resistance [35]. As skeletal muscles are the primary sink for insulin-stimulated glucose disposal, the development of muscular insulin resistance may lead to high circulating glucose retention and glucose intolerance.

The upregulation of RAGE caused by HF/HS diet were effectively prevented by GA treatment as evidenced by the significant downregulation in the aorta, AM and SAT. The downregulation was paralleled with the reduction in the circulating AGEs. This indicates that the AGE-lowering effect of GA might have hindered the RAGE activation. The multiligand functionality of RAGE also suggests multiple inhibitory effects of GA on other ligands. Indeed, GA can inhibit HMGB1 secretion, resulting in the reduction of a series of inflammatory and oxidative markers in a dose-dependent manner [36]. These inhibitory properties of GA on different RAGE ligands may effectively prevent diet-induced RAGE activation and overexpression which then help to attenuate the progression of cellular injury due to metabolic derangements. Although GA treatment could lower serum AGE level and impede the diet-induced upregulation of RAGE, it did not abolish the basal expression of RAGE. This is reflected in the observation that the rats on normal diet and the rats on HF/HS diet and GA treatment shared comparable RAGE expression across all the studied tissues. This is essential as the physiological functions of RAGE, which is associated with inflammatory response and innate immunity [37], may be preserved despite the GA treatment.

## Experimental Section

### Source of the Natural Product

Ready-to-use GA ammonium salt from G*lycyrrhiza* root (licorice) was purchased in solid powder form from Sigma–Aldrich (Selangor, Malaysia). The compound is extracted by HPLC. The product number is G2137. More information about the compound is available at the company website [38].

### General Experimental Procedures

The rats were obtained from the Monash University Animal Facility unit. Standard rat chow, coarse granulated cane sugar and ghee were purchased from Gold Coin, Malayan Sugar Manufacturing and Michelle Foods Sdn. Bhd. whose companies are all based in Malaysia. Glucose oxidase and peroxidase used in Trinder’s glucose oxidase test were purchased from Sigma–Aldrich (Selangor, Malaysia). Rat/Mouse Insulin ELISA kit was purchased from Millipore (Selangor, Malaysia). Rat Glycated Haemoglobin A1c (GHbA1c) ELISA Kit was purchased from CUSABIO^®^ (Wuhan, China) while OxiSelect™ Advanced Glycation End Product (AGE) ELISA Kit was acquired from Cell Biolabs (Selangor, Malaysia). Total RNA extraction kit such as Qiagen RNeasy Mini Kit, Qiagen RNeasy Lipid Tissue Mini Kit and Qiagen Omniscript Reverse Transcription kit were obtained from Qiagen (Kuala Lumpur, Malaysia).

### Animal Sampling and Treatment

The use and handling of animals in the research project have been approved by Monash University School of Biomedical Sciences Animal Ethics Committee (AEC Approval Number: MARP/2012/043). All applicable institutional and/or national guidelines for the care and use of animals were followed. Twenty four male, eight-week old Sprague–Dawley rats (*Rattus norvegicus*) with an initial weight of around 160–200 g were obtained. Throughout the entire experiment, the rats were kept individually in polypropylene cages with paper bedding at 23 ± 1 °C and 12-hour-light-and-dark exposure (lights on at 0600 and lights out at 1800). Prior to treatment, the rats were acclimatized for 1 week to allow them to adapt to the laboratory conditions. During that time, they were given standard rat chow and tap water. After the acclimatization, the rats were randomised into three groups: groups A, B and C (n = 8 per group). Group A rats were provided standard rat chow while Groups B and C rats were on/diet (30 % ghee and 30 % sugar). The HF/HS diet was prepared by evenly mixing powdered standard rat chow, coarse granulated cane sugar and ghee in a ratio of 4:3:3 respectively. The mixture was then baked for 10 min at 160 °C and stored at 4 °C in a fridge until use. The therapeutic intervention, 100 mg/kg GA was subjected exclusively for group C rats via oral administration in drinking water. The treatment duration was 28 days.

### Sample Collection

After the treatment period had completed, the rats were subjected to 8 h fasting before being humanely sacrificed under the effects of 75 mg/kg of ketamine and 10 mg/kg of xylazine hydrochloride via intraperitoneal injection. Whole blood, red blood cell lysate and serum were collected. Red blood cell lysate and serum were stored at −80 °C until further use. The liver, kidney, aorta, heart, AM, QF, SAT and VAT were harvested promptly, snap frozen in liquid nitrogen and stored at −80 °C.

### Biochemical Tests

Blood glucose was measured using Trinder’s glucose oxidase test while serum insulin was determined using based Rat/Mouse Insulin ELISA Kit. To quantify insulin resistance, HOMA-IR was computed based on the fasting blood glucose and fasting insulin levels according to the formula as shown [39].

$$\text{HOMA}-\text{IR}=\frac{Fasting\;blood\;glucose\;(mmol/L)\times Fasting\;serum\;insulin\;(mU/L)}{22.5}$$

HbA1c concentrations were determined using competitive-based Rat Glycated Haemoglobin A1c (GHbA1c) ELISA Kit. Serum AGE levels were measured using OxiSelect™ Advanced Glycation End Product (AGE) ELISA Kit.

### RNA Extraction and cDNA Synthesis

Total RNA was extracted from the liver, kidney, heart, aorta, AM and QF using the Qiagen RNeasy Mini Kit while that of the SAT and VAT were isolated using the Qiagen RNeasy Lipid Tissue Mini Kit. The concentration and purity of the extracted RNA was checked by measuring the absorbance at 260 and 280 nm. RNA integrity was assessed using agarose gel electrophoresis. RNase-free DNase treatment was carried out using Fermentas DNase I RNase-free (Fermentas, Malaysia) to avoid genomic DNA contamination. RNA extracts were then used as the template for cDNA synthesis with Qiagen Omniscript Reverse Transcription kit.

### Quantitative RT-PCR of RAGE

Rotor-Gene Q (Qiagen, USA) was used for qRT-PCR. RAGE was the gene of interest while β-actin gene (BAC) was the chosen endogenous housekeeping gene for normalization of the gene of interest during comparison. The primers and probes are outlined in Table 1. The comparisons of RAGE expression between groups were performed using Relative Expression Software Tool for Rotor-Gene 3000 and 6000 (REST-RG©) Version 3. Agarose gel electrophoresis was carried out to confirm the specificity of the qRT-PCR products.

**Table 1:** Nucleotide sequences of RAGE and BAC primers and probes

Primer/probe	Nucleotide sequence (5′ → 3′)
RAGE forward primer	CCC TGA CCT GTG CCA TCT CT
RAGE reverse primer	GGG TGT GCC ATC TTT TAT CCA
RAGE probe	[6FAM] CCC AGC CTC CCC CTC AAA TCC A [BHQ1]
BAC forward primer	GTA TGG GTC AGA AGG ACT CC
BAC reverse primer	GTT CAA TGG GGT ACT TCA GG
BAC probe	[TET] CCT CTC TTG CTC TGG GC [BHQ1]

### Statistical Analysis

All data were processed and analysed using Statistical Package for the Social Sciences (SPSS) Version 20.0 except relative expressions for RAGE which were analysed using REST-RG© Version 3. For parametric data, one-way ANOVA was used to make comparisons between groups followed by Post Hoc (Tukey) test while for non-parametric data, Kruskal–Wallis test was used followed by Mann–Whitney *U* test. To analyse the relative expressions of RAGE, take-off points and PCR reaction efficiencies of each sample were imported from ‘Comparative Quantitation’ feature of Rotor-Gene Q into the REST-RG© Version 3 where the expression ratios of target (treatment group) to calibrator (control group) were calculated. For all data analyses, a *p* value of ≤ 0.05 was considered statistically significant.

## Conclusion

In consistence with previous findings reported by Chandramouli et al. [9], HF/HS diet could lead to impaired glucose tolerance and insulin resistance which were effectively prevented by GA treatment at a dosage of 100 mg/kg/day. GA treatment could also significantly reduce circulating AGEs by a mechanism which was believed to be independent from its anti-hyperglycaemic effect. Furthermore, HF/HS diet triggered significant RAGE overexpression in the AM. RAGE upregulation which did not reach statistical significance was also detected in the heart, aorta and SAT. GA treatment managed to prevent the diet-induced RAGE expression dysregulation in the aorta, AM and SAT without jeopardising the basal expression in all the studied tissues. Thus, GA can effectively offset the negative impacts on the metabolism caused by HF/HS diet. Its inhibitory effects on the AGE-RAGE axis also make it a potential treatment for diabetic vascular diseases aside from being a preventive drug for the metabolic syndrome.
